# BLM SUMOylation regulates ssDNA accumulation at stalled replication forks

**DOI:** 10.3389/fgene.2013.00167

**Published:** 2013-09-04

**Authors:** Karen J. Ouyang, Mary K. Yagle, Michael J. Matunis, Nathan A. Ellis

**Affiliations:** ^1^Department of Medicine, University of ChicagoChicago, IL, USA; ^2^Department of Pediatrics, Institute of Human Genetics, University of Illinois at ChicagoChicago, IL, USA; ^3^Department of Biochemistry and Molecular Biology, Bloomberg School of Public Health, Johns Hopkins UniversityBaltimore, MD, USA

**Keywords:** Bloom′s syndrome, DNA repair foci, homologous recombination, RecQ DNA helicases, replication fork stability

## Abstract

Polymerase stalling results in uncoupling of DNA polymerase and the replicative helicase, which generates single-stranded DNA (ssDNA). After stalling, RAD51 accumulates at stalled replication forks to stabilize the fork and to repair by homologous recombination (HR) double-strand breaks (DSBs) that accumulate there. We showed recently that SUMO modification of the BLM helicase is required in order for RAD51 to accumulate at stalled forks. In order to investigate how BLM SUMOylation controls RAD51 accumulation, we characterized the function of HR proteins and ssDNA-binding protein RPA in cells that stably expressed either normal BLM (BLM+) or SUMO-mutant BLM (SM-BLM). In HU-treated SM-BLM cells, mediators BRCA2 and RAD52, which normally substitute RAD51 for RPA on ssDNA, failed to accumulate normally at stalled forks; instead, excess RPA accumulated. SM-BLM cells also exhibited higher levels of HU-induced chromatin-bound RPA than BLM+ cells did. The excess RPA did not result from excessive intrinsic BLM helicase activity, because *in vitro* SUMOylated BLM unwound similar amounts of replication-fork substrate as unSUMOylated BLM. Nor did BLM SUMOylation inhibit binding of RPA to BLM *in vitro*; however, in immunoprecipitation experiments, more BLM-RPA complex formed in HU-treated SM-BLM cells, indicating that BLM SUMOylation controls the amount of BLM-RPA complex normally formed at stalled forks. Together, these results showed that BLM SUMOylation regulates the amount of ssDNA that accumulates during polymerase stalling. We conclude that BLM SUMOylation functions as a licensing mechanism that permits and regulates HR at damaged replication forks.

## Introduction

DNA replication is a fundamental process in all living organisms in which the genetic material is duplicated. Highly regulated checkpoint and DNA repair mechanisms ensure that the genome is faithfully replicated with each round of cell division. In mammalian cells, homologous recombination (HR) is an essential repair mechanism that stabilizes damaged DNA replication forks, repairs double-strand breaks (DSBs) that occur during DNA replication, and helps restore productive DNA synthesis following disruption or breakage of replication forks. Although HR is critical in maintaining genome integrity during replication, it is tightly regulated to avoid harmful outcomes.

In the autosomal recessive, clinical entity Bloom's syndrome (BS), genome integrity is strikingly destabilized due to null mutations in the gene *BLM* (Ellis et al., 1995). The BLM protein is an ATP-dependent DNA helicase of the RecQ family, and it possesses multiple functions in DNA replication and HR (Lambert et al., 2010; Wechsler et al., 2011). BLM is one of the first components to be recruited to sites of DNA replication after treatment with agents that inhibit fork progression where it is thought to stabilize stalled forks (Davalos and Campisi, 2003; Sengupta et al., 2003, 2004). In HR-mediated DSB repair, BLM together with exonucleases EXO1 and DNA2 promote resection of DSBs, generating 3′ single-stranded DNA (ssDNA) tails that provide a substrate for loading of the RAD51 recombinase (Nimonkar et al., 2008, 2011; Mimitou and Symington, 2009). BLM preferentially unwinds substrates that resemble recombination intermediates, such as X-junctions and D-loops, and it is a member of a complex, which includes TopIIIα, BLAP75, and BLAP18 that possesses the unique capacity to dissolve a late recombination intermediate, the double Holliday junction, such that only non-crossover products are generated (Wu and Hickson, 2003; Raynard et al., 2006; Wu et al., 2006; Bussen et al., 2007; Singh et al., 2008; Xu et al., 2008). This activity is also important at sites of termination of DNA replication, because BLM accumulates on late replication intermediates to assist in duplex separation so that DNA replication can be efficiently completed (Chan et al., 2007, 2009; Lukas et al., 2011; Barefield and Karlseder, 2012).

BS cells, which lack BLM activity, display numerous characteristics that are the consequence of excessive HR, including high rates of loss of heterozygosity (Langlois et al., 1989; Groden et al., 1990; LaRocque et al., 2011), excessive chromosome abnormalities, such as telomere fusions, ring chromosomes, and quadriradial chromosomes (German, 1964; German and Crippa, 1966), and a high rate of sister chromatid exchange (SCE)(Chaganti et al., 1974). In addition, BS cells exhibit defects in DNA replication that might lead to excess HR, including accumulation of abnormal DNA replication intermediates (Lönn et al., 1990; Li et al., 2004), slower than normal DNA-chain growth (Hand and German, 1975; Rao et al., 2007), and abnormal origin firing (Davies et al., 2007). BS cells are hypersensitive to replication inhibitors such as hydroxyurea (HU)(Davies et al., 2004), and replication forks in BS cells recover inefficiently from HU-induced stalling, exhibiting accelerated accumulation of DSBs after release (Davies et al., 2007; Ouyang et al., 2009; Sidorova et al., 2013).

In our previous work, we showed that BLM undergoes post-translational modification at lysines K317 and K331 by SUMO-1 and SUMO-2 (Eladad et al., 2005); cells that expressed a GFP-BLM that was mutated at these two SUMO-acceptor sites, which we refer to as SUMO-mutant BLM (SM-BLM) cells, exhibited impairments of replication-associated HR, as evidenced by increased HU-induced DNA damage and reduced HU-induced SCE (Ouyang et al., 2009). We further showed that SM-BLM cells have a defect in the recruitment or retention of the RAD51 recombinase at stalled replication forks and that SUMOylation of BLM *in vitro* increased the binding efficiency between BLM and RAD51, suggesting that BLM SUMOylation could act as a switch to turn on BLM's function in HR repair of stalled forks (Ouyang et al., 2009). Although our data indicates that SUMOylation of BLM regulates the recruitment or retention of RAD51 at stalled replication forks, the mechanism underlying the defect in RAD51 localization in SM-BLM cells remains unclear.

Replication inhibitors such as HU cause stalling of replicative polymerases on DNA, uncoupling of the replicative helicase from polymerases, and the generation of excess ssDNA (Byun et al., 2005). After HU treatment, excess ssDNA is detectable within minutes as evidenced by the accumulation of focal concentrations of ssDNA binding protein—replication protein A (RPA)—at stalled replication forks (Balajee and Geard, 2004; Petermann et al., 2010). In the HR pathway, RAD51 is normally loaded onto RPA-bound ssDNA by a process that involves mediators (e.g., BRCA2 and RAD52) that substitute RAD51 for RPA on ssDNA (Heyer et al., 2010); however, after treatment of normal cells with HU, RAD51 does not accumulate at stalled replication forks for several hours (Saintigny et al., 2001; Petermann et al., 2010). We hypothesize that a licensing mechanism exists that controls the loading of RAD51 onto ssDNA at stalled replication forks. The licensing mechanism could prevent premature loading of RAD51 at stalled replication forks and activate HR after fork breakage. To explain the deficit of RAD51 loading at stalled forks in SM-BLM cells, we hypothesized that SUMOylation of BLM is a key step in the licensing mechanism. To test this hypothesis, we analyzed the function of mediators and RPA at stalled forks in HU-treated SM-BLM cells.

## Results

### Mediator accumulation at stalled replication forks is impaired in SUMO-mutant BLM cells

**Figure 1:**
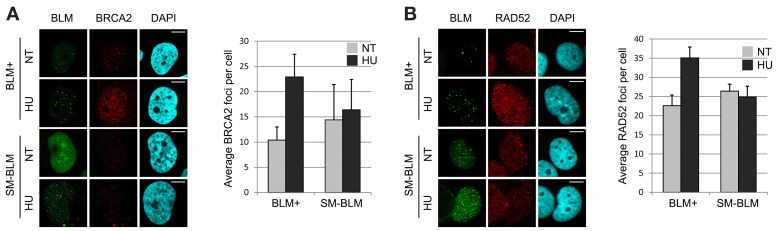
**Mediator accumulation at stalled replication forks is impaired in SUMO-mutant BLM cells. (A)** Immunofluorescence images of BLM+ and SM-BLM cells untreated (NT) or treated with 0.5 mM HU for 24 h (HU) and stained for BRCA2, with graphical representation of mean numbers of BRCA2 foci. **(B)** Same as **(A)** but stained for RAD52, with graphical representation as in **(A)**. Two independent experiments were carried out on each of two BLM+ and two SM-BLM clones. Error bars represent the standard deviations of the results of each experiment. After treatment with HU, there were significantly more BRCA2 and RAD52 foci in BLM+ cells (*p* < 0.001 for both comparisons) but not in SM-BLM cells (*p* = 0.64 for BRCA2 and *p* = 0.38 for RAD52). *P*-values were calculated using mixed effects linear models as described in Materials and Methods. Bars indicate 10 μm.

Because the loading mechanism of RAD51 onto RPA-bound ssDNA requires recruitment of mediators to the repair site (Thorslund et al., 2010), we tested whether the mediators BRCA2 and RAD52 localized normally in HU-treated SM-BLM cells. As expected, treatment of BLM+ cells with 0.5 mM HU for 24 h induced an increase in BRCA2 foci from 10.4 foci/cell to 22.9 foci/cell. On the contrary, despite the presence of approximately twice the number of γ-H2AX and BLM foci, untreated SM-BLM cells exhibited 14.4 BRCA2 foci/cell and HU treatment failed to induce a significant increase in BRCA2 foci to 16.4 foci/cell (Figure 1A). Similarly, whereas HU treatment of BLM+ cells induced an increase in RAD52 foci from 22.6 foci/cell to 35.1 foci/cell, HU treatment of SM-BLM cells resulted in virtually no change in RAD52 foci (from 26.4 foci/cell in untreated cells to 24.9 foci/cell in HU-treated cells; Figure 1B). These data suggested that the impairment of RAD51 localization in SM-BLM cells was explained by an upstream defect in mediator accumulation at stalled forks.

### Excess RPA accumulates at stalled replication forks in SUMO-mutant BLM cells

**Figure 2:**
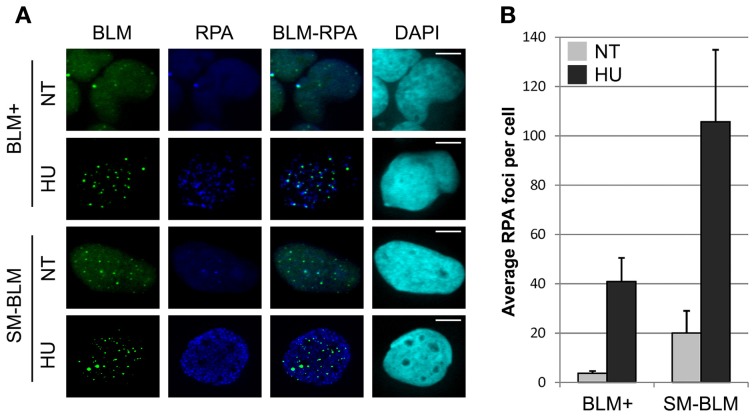
**Excess RPA accumulates at stalled replication forks in SUMO-mutant BLM cells. (A)** Immunofluorescence images of BLM+ and SM-BLM cells untreated (NT) or treated with 0.5 mM HU for 24 h (HU) and stained for RPA. Bars indicate 10 μm. **(B)** Graphical representation of mean numbers of RPA foci. Three independent experiments were carried out for each of two BLM+ and two SM-BLM clones. Error bars represent the standard deviations of the results of each experiment. There were significantly more RPA foci in SM-BLM cells compared to BLM+ cells (*p* < 0.001) and HU treatment induced significantly more RPA foci in both cell lines (*p* < 0.001). Interaction was also observed indicating that the difference in effect of HU treatment on the two cell lines is significant (*p*_int_ = 0.002). *P*-values were calculated using mixed effects linear models as described in Materials and Methods.

Because accumulation of BRCA2 and RAD52 mediators at stalled forks was impaired after HU treatment, we reasoned that the impairment could be caused by less ssDNA accumulation after HU treatment. To test this possibility, we compared the accumulation of RPA at stalled forks in SM-BLM and BLM+ cells. We treated cells with or without 0.5 mM HU for 24 h, then quantified RPA foci by scoring the number of RPA foci/cell (Figure 2A). Untreated SM-BLM cells exhibited a higher number of RPA foci than untreated BLM+ cells (21.1 foci/cell vs. 3.7 foci/cell, respectively). HU induced RPA foci in both SM-BLM and BLM+ cells (105.8 foci/cell vs. 40.5 foci/cell, respectively; Figure 2B). The absolute increase was 84.7 foci/cell in SM-BLM cells compared to 36.8 foci/cell in BLM+ cells. We noted that in HU-treated conditions in both SM-BLM and BLM+ cells, BLM co-localized to a high degree with a subset of RPA foci. These results ruled out the possibility that BLM SUMOylation is required for ssDNA accumulation; on the contrary, SM-BLM cells contained a vast excess of RPA foci in both untreated and HU-treated conditions. These data indicated that more ssDNA accumulated at stalled replication forks in SM-BLM compared to BLM+ cells.

### Excess chromatin-bound RPA in SM-BLM cells

**Figure 3:**
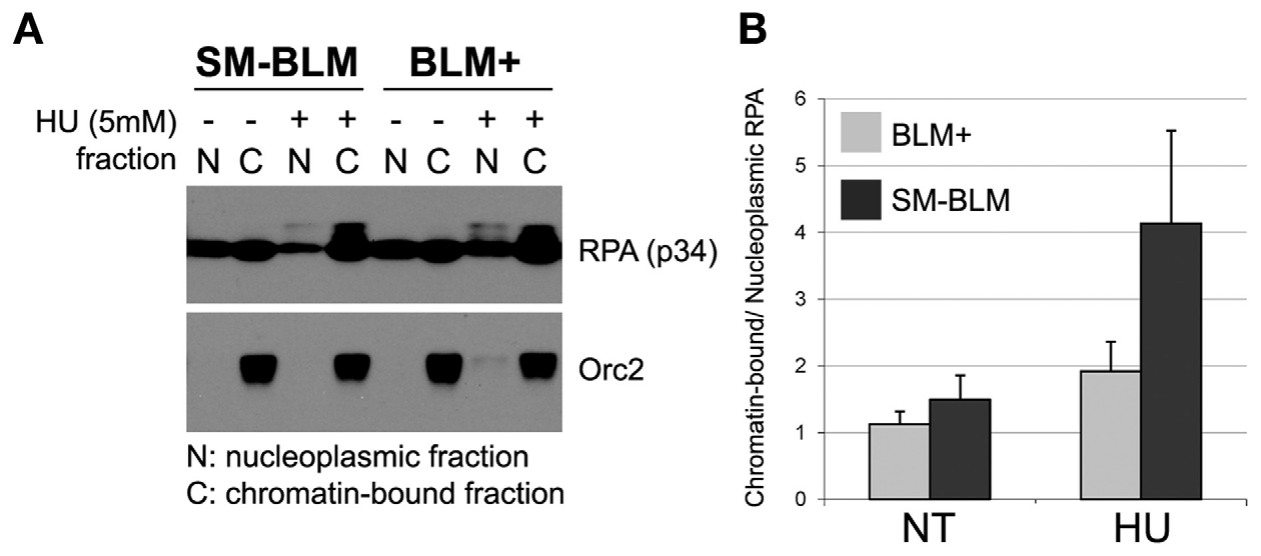
**Increased levels of RPA accumulate on chromatin in HU-treated SM-BLM cells**. BLM+ and SM-BLM cells were untreated or treated with 5 mM for 6 h. **(A)** Chromatin and nucleoplasmic fractions were isolated from cells and solubilized RPA was analyzed by immunoblot using antibodies to the p34 subunit. Orc2 was used for quality control of the preparation of the chromatin-bound fraction. **(B)** Graph depicting the ratio of chromatin-bound RPA to nucleoplasmic RPA. A minimum of two independent experiments were carried out on each of two BLM+ and two SM-BLM clones. Error bars represent the standard deviations of the combined data.

Because SM-BLM cells accumulated excess RPA foci, we predicted that more RPA would be bound to chromatin. To test this prediction, we isolated chromatin and nucleoplasmic fractions from BLM+ and SM-BLM cells untreated or treated with 5 mM HU for 6 h and analyzed extracted proteins by immunoblot. In untreated conditions, the ratio of chromatin-bound RPA to total RPA was approximately equal in BLM+ and SM-BLM cells. After HU treatment, however, RPA shifted from nucleoplasmic fractions to chromatin-bound fractions in response to replication stress (Figure 3A). The ratio of chromatin-bound RPA to total RPA increased four fold in SM-BLM cells compared to two fold in BLM+ cells (Figure 3B). The increased amount of chromatin-bound RPA after HU treatment in SM-BLM cells was consistent with the indirect immunofluorescence data that showed the accumulation of excess RPA foci. Together, these data indicated that BLM SUMOylation functions to limit the formation of excess RPA-ssDNA complex at stalled replication forks.

### DNA helicase activity of SUMOylated BLM is normal

**Figure 4:**
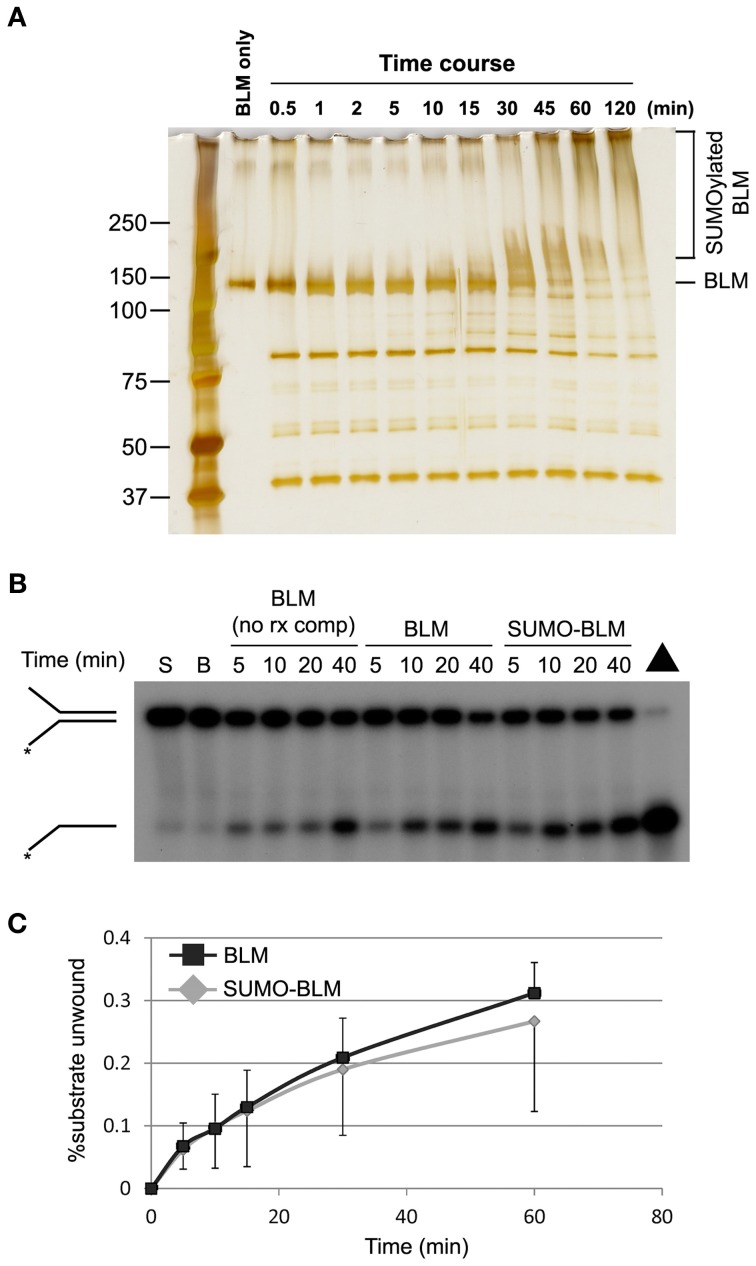
**DNA helicase activity of SUMOylated BLM is similar to unSUMOylated BLM. (A)** BLM SUMOylation reactions were carried out, time points were removed, and the reactions were stopped with sample buffer. The reaction products were analyzed by sodium dodecyl sulphate-polyacrylamide gel electrophoresis followed by silver staining. **(B)** SUMOylated (SUMO-BLM) or unSUMOylated BLM (BLM) was prepared by incubating 200 ng of purified recombinant BLM with SUMO reaction components in the presence (to make SUMOylated BLM) or absence (to make unSUMOylated BLM) of ATP. Helicase assays were performed by incubating the SUMO reaction products with ^32^P-labeled synthetic replication-fork substrate, and the helicase reactions were stopped at different times. The products were analyzed by non-denaturing polyacrylamide gel electrophoresis followed by autoradiography. The position of the forked duplex and the unwound product are indicated on the left. S, unreacted substrate; B, beads only with unreacted substrate; Δ, boiled substrate; BLM (no rx comp), helicase reactions performed with BLM protein that was not exposed to SUMO reaction components as a positive control. **(C)** Graph showing quantification of the percentage of unwound substrate by SUMOylated and unSUMOylated BLM prepared in these experiments by nickel-NTA pull down. A minimum of two DNA unwinding experiments were performed for each method of BLM SUMOylation preparation shown in **(B)** and **(C)**.

The excessive accumulation of fork-associated RPA raised the possibility that excess ssDNA was generated at stalled forks in SM-BLM cells due to a failure to suppress BLM's DNA unwinding activity at the fork. To test this hypothesis, we compared the DNA helicase activity of SUMOylated and unSUMOylated BLM *in vitro*. Purified His-tagged BLM helicase was SUMOylated *in vitro* using purified human E1, UBC9, and SUMO-2 in a reaction that required ATP. After incubation of the BLM with reaction components for 2 h, >95% of the BLM was SUMOylated, and multiple moieties of SUMO-2 were attached to most of the BLM (Figure 4A). We then compared helicase activity of the SUMOylated and unSUMOylated BLM in two ways: (1) BLM SUMOylation reactions were prepared with or without ATP and reaction products were added directly to helicase assays; or, (2) SUMOylated and unSUMOylated BLM was partially purified from the SUMO reaction components by pull-down on nickel-NTA beads, after which bead-bound BLM was added to helicase assays. BLM activity was assayed on a ^32^P-labeled DNA replication-fork-like substrate (38-nucleotide duplex DNA with two 12-nucleotide ssDNA tails) and percent unwinding was measured by electrophoresis through non-denaturing polyacrylamide gels followed by autoradiography. In both types of experiment, the percent of DNA substrate unwound over time was indistinguishable for SUMOylated compared to unSUMOylated BLM (Figures 4B,C). These results showed that BLM SUMOylation does not function to suppress BLM's intrinsic DNA helicase activity. Therefore, the excessive accumulation of fork-associated RPA in SM-BLM cells was not the result of failure to suppress BLM's intrinsic DNA helicase activity.

### Excess BLM-RPA complex forms in SM-BLM cells

**Figure 5:**
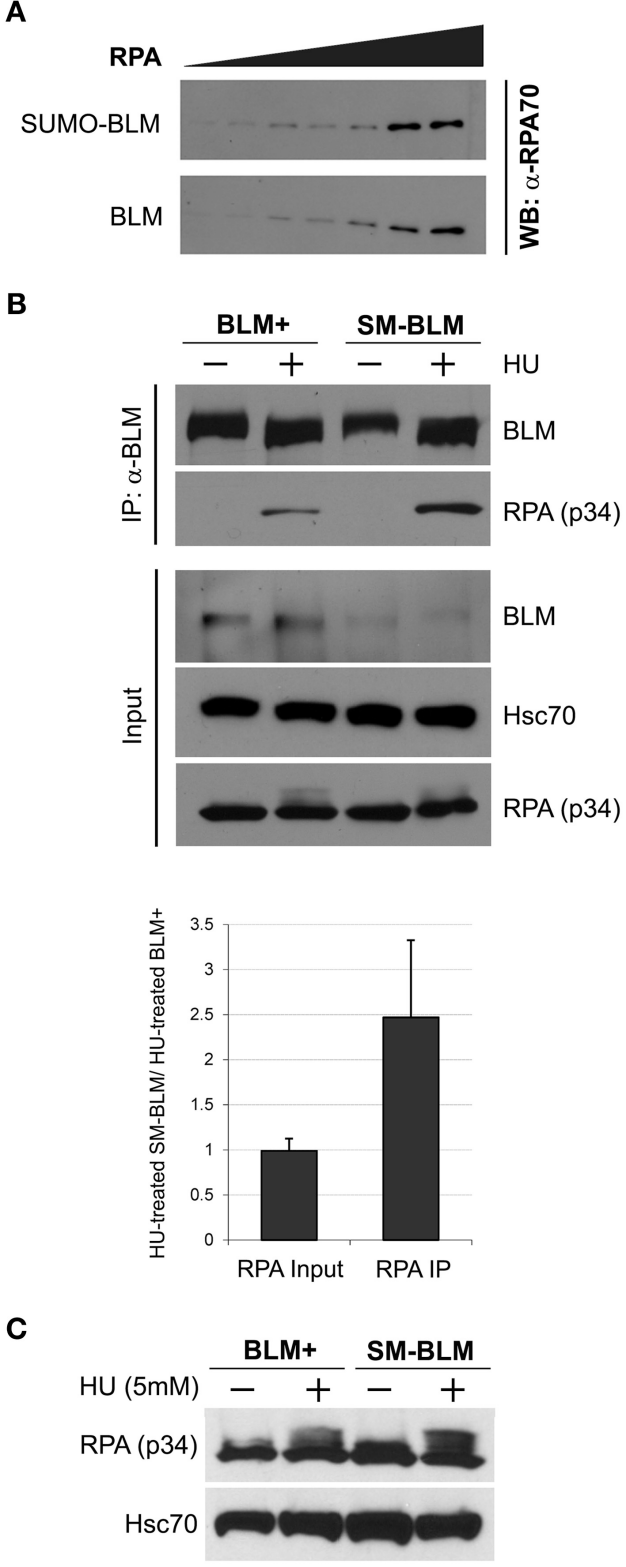
**Excess BLM-RPA complex forms in SM-BLM cells. (A)** SUMOylation of BLM did not affect BLM-RPA binding *in vitro*. SUMOylated (SUMO-BLM) or unSUMOylated (BLM) BLM were bound to Ni-NTA beads and incubated with increasing amounts of purified human RPA protein for 30 min at room temperature. The beads were washed three times. Bound RPA protein was analyzed by immunoblot with anti-RPA/p70 antibody. A bead-only experiment showed that no excess RPA bound to beads in the absence of BLM. **(B)** BLM binds more RPA in SM-BLM cells than BLM+ cells. Cells were untreated or treated with 5 mM HU for 6 h. Immunoprecipitation of RPA was carried out using antibodies to the GFP tag on GFP-BLM. Input consisted of 2% of the initial cell extracts. Hsc70 was used as a loading control. A minimum of two independent experiments were carried out for each of two BLM+ and two SM-BLM clones. The graph depicts the ratio of input and immunoprecipitated RPA in HU-treated SM-BLM cells over HU-treated BLM+ cells. **(C)** HU does not induce significantly different levels of RPA and phosphorylated RPA in SM-BLM cells compared to BLM+ cells. Cells were untreated or treated with 5 mM HU for 6 h. Whole cell lysates were extracted and analyzed by immunoblot with antibodies to the p34 subunit of RPA. Hsc70 was used as a loading control. A minimum of two independent experiments were carried out for each of two BLM+ and of two SM-BLM clones. Error bars represent the standard deviations of the combined data.

BLM interacts with the 70 kD subunit of RPA (RPA-70), and RPA stimulates BLM's DNA helicase activity (Brosh et al., 2000). If BLM SUMOylation functions to inhibit BLM-RPA interaction, then excess RPA accumulation in SM-BLM cells could result from a failure to suppress BLM-RPA interaction. To test this hypothesis, we compared the binding of purified RPA to SUMOylated or unSUMOylated BLM *in vitro*. BLM SUMOylation reactions were prepared with or without ATP, reaction products were mixed with RPA, and BLM-RPA complexes were pulled down with nickel-NTA beads. The amount of BLM-bound RPA was then measured by immunoblot analysis with anti-RPA/p70 antibodies. In these assays, the amount of BLM-bound RPA-70 detected was the same whether or not BLM was SUMOylated (Figure 5A). These data suggested that BLM SUMOylation does not directly affect interaction between BLM and RPA.

Because BLM SUMOylation could affect BLM and RPA interaction indirectly, we compared BLM-RPA complex formation *in vivo* by immunoprecipitation of BLM from HU-treated and untreated BLM+ vs. SM-BLM cells. In untreated cells, little to no detectable RPA was pulled down with BLM in either cell line. However, in HU-treated cells, more RPA was pulled down with BLM in SM-BLM cells compared to BLM+ cells (Figure 5B). Finding increased BLM-RPA complex formation in SM-BLM cells correlated with the excessive accumulation of RPA foci in SM-BLM cells, and it suggested that BLM SUMOylation has a role in preventing the accumulation of excess ssDNA at stalled forks.

To test whether the increased BLM-RPA complex formation in SM-BLM cells was trivially a consequence of higher levels of RPA expression in these cells, we performed immunoblot analysis of untreated and HU-treated SM-BLM and BLM+ cells. Analysis of total cell extracts indicated that SM-BLM and BLM+ cells contained similar amounts of RPA protein with or without treatment with HU (Figure 5C). Together with the *in vitro* data, the results suggested that BLM SUMOylation affects BLM-RPA complex formation indirectly, possibly through the mediation of other proteins at the fork. We concluded that BLM SUMOylation regulates BLM-RPA interaction at stalled forks.

In these experiments, we noted that HU-induced RPA phosphorylation did not differ significantly between BLM+ and SM-BLM cells, despite the presence of excess RPA at stalled forks in SM-BLM cells (Figure 5C).

## Discussion

The data presented here demonstrated that BLM SUMOylation regulates RPA and mediator accumulation at stalled replication forks, limiting the generation of excess ssDNA there. In HU-treated SM-BLM cells, a large excess of ssDNA accumulated at stalled forks, as evidenced by excess RPA foci and greater amounts of chromatin-bound RPA. With so much ssDNA generated at stalled forks in SM-BLM cells, we would expect large accumulations at the fork of both mediators BRCA2 and RAD52, and RAD51 as well. On the contrary, these proteins did not accumulate normally at stalled forks. These two observations are therefore evidence that BLM SUMOylation is necessary to license the HR mechanism at stalled forks, as mediator-dependent RAD51 complexes did not form on RPA-coated ssDNA in its absence. Because RAD51 loading onto ssDNA depends on mediator function, our results explain why RAD51 is not recruited to stalled forks in SM-BLM cells; however, the mechanism explaining why mediators are not recruited is still enigmatic.

The uncoupling of DNA replication from the replicative helicase during fork stalling can lead to extended stretches of ssDNA and later to replication-associated DSBs that could be processed by exonucleases (Petermann and Helleday, 2010). Because RPA-ssDNA complexes form on both of these cellular substrates, we do not know a priori whether BLM SUMOylation is important before or after the replication fork breaks. In our previous work (Ouyang et al., 2009), we showed that 24-h HU treatment of SM-BLM cells did not lead to increased DSBs in comparison to untreated SM-BLM cells. Moreover, the effect of BLM SUMOylation on RAD51 accumulation at stalled forks was apparent after a 1-h HU treatment of SM-BLM cells before DSBs have had a chance to accumulate (Saintigny et al., 2001; Petermann et al., 2010). Therefore, these data suggest that BLM SUMOylation is important in the generation of ssDNA after helicase-polymerase uncoupling and before fork breakage.

Formally speaking, the function of BLM SUMOylation in HR licensing at the fork could result from either positive or negative regulatory effects or both. Previous work has shown that RPA interacts with BLM, inhibiting BLM's strand annealing activity while increasing the efficiency of its helicase activity (Brosh et al., 2000; Doherty et al., 2005; Bartos et al., 2006). SUMOylation did not inhibit BLM-RPA interaction *in vitro*, yet more BLM-RPA complex formed in SM-BLM cells than in BLM+ cells after replication stress. Intriguingly, RPA is SUMOylated by SUMO-2 after treatment with camptothecin (CPT), which generates replication-associated DSBs (Dou et al., 2010). Cells that express a SUMO-mutant RPA have defects in RAD51 accumulation and DSB repair at IR-induced DSBs and CPT-induced broken forks; but increased RPA SUMOylation was not reported to occur during replication stress with UV or HU treatment (Dou et al., 2010). Thus, SUMOylation of BLM and RPA seem to have similar effects on the accumulation of RAD51, one in the context of replication fork stalling and the other in the context of replication fork breakage. Further studies are needed to examine the role of SUMOylation on the interaction of RPA and BLM and the possible effects of SUMOylation on each protein's biochemical activities.

In normal cells, the amount of ssDNA that accumulates after replication stalling is regulated by damage sensing and checkpoint pathways that recruit proteins to stabilize the fork and inhibit further unwinding (Sleeth et al., 2007). Because BLM protein is recruited to the fork immediately after stalling occurs, it is possible that pathologically unrestricted SM-BLM protein generates excessive DNA unwinding at the fork. We showed that BLM helicase activity is not inhibited by SUMOylation; therefore, if SUMOylation prevents BLM from unwinding DNA at the fork, it must do so via interaction with another protein. Because RPA stimulates BLM helicase activity, it is possible that SUMOylation negates RPA's stimulatory effect. Alternatively, BLM SUMOylation could have an indirect effect on the accumulation of RPA-ssDNA complex through interaction with ATR-mediated checkpoint signaling.

Another possibility is that BLM SUMOylation activates BLM or another protein's ssDNA annealing activity. A novel ssDNA annealing function was recently identified in the N-terminal portion of BLM distinct from an annealing activity reported for the helicase domain (Chen and Brill, 2010); this region coincides with the region containing the SUMO-acceptor sites and the region required for SUMO binding (Eladad et al., 2005; Zhu et al., 2008). This ssDNA annealing activity of BLM could function to stabilize stalled forks by reannealing excess ssDNA. The annealing helicase HARP (HepA-related protein) has a role in ssDNA annealing that leads to stabilization of stalled forks (Bansbach et al., 2009; Driscoll and Cimprich, 2009; Yuan et al., 2009), and BLM SUMOylation could de-repress or directly promote the activities of these proteins (Yusufzai and Kadonaga, 2008, 2010; Sen et al., 2012).

Many key components and functions of the HR machinery are highly conserved. An exception to this rule lies with mediator proteins. While Rad52 is an essential component in yeast HR (Pâques and Haber, 1999), RAD52 gene knockout in mice shows no significant effect on cell viability or on HR and DNA repair capacities (Rijkers et al., 1998; Yamaguchi-Iwai et al., 1998). Instead, the BRCA2 mediator protein, a component that is not present in budding yeast, plays a central role in mammalian DSB repair by HR (Xia et al., 2001; Yang et al., 2005; Carreira et al., 2009). Recent work has shown that RAD52 inactivation is synthetically lethal with BRCA2 deficiency in human cell lines, indicating overlapping functions between RAD52 and BRCA2 in RAD51-dependent HR repair (Feng et al., 2011). Some have suggested that RAD52's primary function is not in the repair of DSBs with two broken ends but in the protection and repair of stalled and broken replication forks (Wray et al., 2008; Feng et al., 2011). Our results showed that SM-BLM cells have a defect in the accumulation of both RAD52 and BRCA2 at stalled forks, indicating that BLM SUMOylation is necessary for efficient mediator recruitment to stalled replication forks. We note that RAD52 is SUMOylated by the MMS21 E3 ligase, which is part of the SMC5/6 complex, and RAD52's SUMOylation is required for its normal repair function (Sacher et al., 2006; Santa Maria et al., 2007; Torres-Rosell et al., 2007; Ohuchi et al., 2008; Altmannova et al., 2010). Recent work has indicated that mediators, RAD51, and the Fanconi anemia complex have functions in replication fork stabilization that are independent of their roles in DSB repair (Schlacher et al., 2011, 2012; Feng and Zhang, 2012). RAD52 has ssDNA annealing activity that could help stabilize the replication fork (Wu et al., 2008; Grimme et al., 2010); consequently, the failure to recruit mediators to stalled forks in SM-BLM cells could explain the accumulation of excess RPA-ssDNA, if mediators are required for ssDNA annealing at the fork, or it could be an independent consequence of unSUMOylated BLM activity at the stalled fork.

While excess accumulation of RPA in HU-treated SM-BLM cells could result from either excessive DNA unwinding or impaired ssDNA re-annealing, there is yet another possible explanation relating to replication dynamics. Work in yeast and mammalian cells has shown that replication forks collapsed by prolonged replication stalling do not restart, and replication is rescued instead by new origin firing (Davies et al., 2007; Petermann et al., 2010). In SM-BLM cells, stalled replication forks may collapse more readily, leading to initiation of new forks to compensate for the loss. In this model, the excess RPA foci would be accounted for by activation of dormant replication origins.

The SUMO pathway prevents aberrant recombination events from occurring at damaged replication forks. It positively regulates DSB repair by HR (Liberi et al., 2005; Branzei et al., 2006; Burgess et al., 2007) and it can influence repair pathway choice (Ulrich, 2009; Yang et al., 2011). Multiple components of the HR pathway are SUMOylated, but in most cases a complete mechanistic understanding of SUMO's role in functional regulation of its substrates is lacking. Protein-protein interactions between SUMO binding sites and SUMOs could be important in the recruitment of DNA repair factors such as 53BP1 and BRCA1 to the repair site (Bergink and Jentsch, 2009; Galanty et al., 2009; Morris et al., 2009). Recent work on the SUMO-targeted ubiquitin ligase RNF4 has suggested that SUMOylation could be important for dissociation of proteins such as MDC1 from the repair site (Galanty et al., 2012; Luo et al., 2012; Yin et al., 2012; Vyas et al., 2013), but ubiquitylation by RNF4 could also serve to recruit proteins with ubiquitin-binding sites by making hybrid SUMO-ubiquitin chains (Guzzo et al., 2012). From all these studies, SUMO seems to govern important turnover transitions, promulgating cycles of association and dissociation at the repair site: BLM SUMOylation could both assist in the recruitment of factors like RAD52 and RAD51 and it could also remove BLM from the repair site; both of these roles for SUMOylation would be absent in the SM-BLM protein.

BLM has many functions throughout the processes of DNA replication and HR repair. How these functions are regulated is an important question. SUMOylation is a dynamic process that confers diverse and unique roles to its substrate proteins. The present work shed light on how BLM SUMOylation regulates RPA and mediator functions at stalled forks, and more broadly, how replication forks are maintained under stress when HR is called into play. Our evidence suggests that BLM SUMOylation functions as a licensing mechanism that regulates and permits execution of the HR mechanism at damaged replication forks. The next step is to dissect the specific recruitment and turnover functions of SUMOylation on BLM and other proteins in this licensing mechanism.

## Materials and methods

### Antibodies

For BLM immunoblot analysis, rabbit polyclonal anti-BLM antibodies raised against the first 431 amino acids of human BLM (Beresten et al., 1999) were used. For other immunoblot analyses, rabbit polyclonal anti–γ-H2AX antibodies (Abcam), mouse monoclonal anti-RPA/p34 antibody (Neomarkers), mouse monoclonal anti-RPA/p70 antibody (Neomarkers), rat monoclonal anti-ORC2 antibody (Abcam), and rat monoclonal anti-Hsc70 antibody (Assay Design) were used. Horseradish peroxidase-linked anti-mouse, anti-rabbit (Amersham), and anti-rat (Jackson ImmunoResearch) antibodies were used as secondary antibodies. Agarose conjugated rat monoclonal anti-GFP antibody D153-8 (MBL) was used for immunoprecipitation experiments. For indirect immunofluorescence, we used mouse monoclonal anti–γ-H2AX antibody (Upstate), rabbit polyclonal anti-RAD51 antibodies PC130 (Calbiochem), rabbit polyclonal anti-RAD52 antibodies sc-8250 (Santa Cruz Biotechnology), mouse anti-BRCA2 antibodies 05-666 (Millipore), mouse monoclonal anti-RPA/p34 antibody (Neomarkers), Cy-5–labeled donkey anti-rabbit antibodies (Jackson ImmunoResearch), Alexa Fluor 594-labeled goat anti-mouse, Alexa Fluor 594-labeled goat anti-rabbit, and Alexa Fluor 647-labeled goat anti-mouse antibodies (Invitrogen).

### Immunofluorescence and image analysis

The generation and characterization of BLM+ and SM-BLM cells was described previously (Eladad et al., 2005; Ouyang et al., 2009). Briefly, the SV40-transformed BS fibroblast cell line GM08505 was transfected with normal GFP-BLM or the SM-GFP BLM expression constructs, clones were isolated and selected for analysis that expressed similar amounts of GFP-BLM proteins. For indirect immunofluorescence, BLM+ and SM-BLM cells were seeded on coverslips and then treated with 0.5 mM HU in culture medium for 24 h. At the end of HU treatment, cells were washed and fixed and then stained with anti-RAD51, −BRCA2, −RAD52, or −RPA antibodies, and counterstained with appropriate secondary antibodies labeled with Alexa Fluor (Invitrogen). Fixation and staining was performed as described previously (Eladad et al., 2005; Ouyang et al., 2009). Coverslips were mounted with Prolong Gold antifade reagent containing 4′,6-diamidino-2-phenylindole (DAPI; Invitrogen). Images were captured on a spinning disk confocal microscope (Carl Zeiss, LSM-510), and data were collected using Slidebook 4.1 software. Z-stacks were captured using a 100× oil immersion objective and the optical slice thickness was 0.2 μm. The BRCA2 data was collected on a laser-scanning confocal microscope (Carl Zeiss, LSM-710) and a single plane was imaged.

A focus was defined as a defined area of the nucleus greater than the minimum area of optical resolution (>0.125 μm^2^) in at least one Z-stack in which the fluorescence intensity was greater than the background fluorescence intensity of the nucleoplasm. The maximum number of foci that could be counted in these cells was 150. A typical immunofluorescence experiment consisted of assessment of 30–50 cells per condition. The data presented are from two to three independent experiments performed on two to three clones of each type. Immunofluorescence images for figures were created using Image J and Metamorph software (Molecular Devices).

### Immunoblot analyses

Proteins from cell lysates, chromatin, and nucleoplasmic fractions, and immunoprecipitates were separated on 4–15% sodium dodecyl sulphate (SDS)/polyacrylamide gradient gels (Bio-Rad) and transferred on to polyvinylidene fluoride membranes (Bio-Rad). The membranes were blocked for 1 h at room temperature with Tris-buffered saline (Boston Bioproducts) containing 0.1% Tween 20 and 5% powdered milk (Bio-Rad), washed, and subsequently probed with appropriate primary antibodies either for 2 h at room temperature or overnight at 4°C. The membranes were then incubated with the appropriate horseradish peroxidase-linked secondary antibodies for 1 h at room temperature, washed, and incubated with Western Lightning–ECL, Enhanced Chemiluminescence reagent (Perkin Elmer) for 5 min at room temperature. Proteins labeled with antibodies on the membrane were visualized by detection on film.

### Chromatin isolation

Chromatin preparations were made by the method of Méndez and Stillman (2000). Briefly, cells treated with or without 5 mM HU for 6 h were harvested by centrifugation, washed in PBS, and resuspended in buffer A (10 mM HEPES, pH 7.9, 10 mM KCl, 1.5 mM MgCl2, 0.34 M sucrose, 10% glycerol, 1 mM DTT, 0.1 mM PMSF, and protease inhibitors cocktail). Triton X-100 (0.1%) was added, and the cells were incubated for 5 min on ice. Nuclei were collected in the pellet by low-speed centrifugation at 1300 × g for 4 min at 4°C. Nuclei were washed once in buffer A, and then lysed in buffer B (3 mM EDTA, 0.2 mM EGTA, 1 mM DTT, protease inhibitors cocktail). Nucleoplasmic proteins were separated from chromatin-bound proteins by centrifugation at 1700 × g for 5 min at 4°C. Nucleoplasmic fractions were collected in the supernatant. The chromatin pellet was washed once in buffer B, and centrifuged again under the same conditions. The final chromatin pellet was resuspended in Laemmli sample buffer. HU treatment was performed at 5 mM for 6 h so that BLM quantities would be nearly the same in untreated and treated cells. With respect to the phenotypes studied here, there are no major differences between the 6− and 24-h treatments (Ouyang et al., 2009).

### Immunoprecipitation

Cells treated with or without 5 mM HU for 6 h were harvested, washed twice in PBS buffer, and resuspended in NP-40 lysis buffer (Boston Bioproducts) containing protease inhibitors (Roche) for 30 min. Cell lysates were cleared by centrifugation (15 min, 14,000 × g, 4°C). Cleared cell lysates were then incubated with 20 μl of agarose beads conjugated with anti-GFP antibodies and allowed to mix overnight at 4°C. Proteins bound to beads were separated from the supernatant by centrifugation (3 min, 2000 × g, 4°C) and washed with 5 times volume of lysis buffer. Proteins were extracted from the beads by boiling in Laemmli buffer (Boston Bioproducts) for 5 min and subsequently analyzed by immunoblot.

### SUMOylation of BLM

Two hundred nanograms of His-tagged full-length BLM, purified according to the method of Karow et al. (1997), was SUMOylated in a 50 μl reaction containing 10 mM HEPES (pH 7.3), 110 mM potassium acetate, 2 mM MgCl_2_, 2 mM ATP, and purified recombinant proteins (Zhu et al., 2008), including 200 ng E1, 100 ng UBC9, and 200 ng SUMO-2, at 37°C for increasing amounts of time (0.5, 1, 2, 5, 10, 15, 30, 45, 60, and 120 min). Reactions were stopped by the addition of sample buffer and subsequently analyzed by SDS-polyarcylamide gel electrophoresis followed by Silver staining (Pierce Silver Stain Kit, Thermo Scientific) per the manufacturer's instructions.

### DNA substrate

Oligonucleotides for the fork substrate designed according to Mohaghegh et al. (2001) were purchased from Integrated DNA Technologies. A single oligonucleotide was 5′-end-labeled with [γ−^32^P]ATP (PerkinElmer Life Sciences) using T4 polynucleotide kinase in PNK buffer at 37°C for 30 min per the manufacturer's instructions (New England Biolabs). The labeled substrate was purified using QIAquick Nucleotide Removal kit (Qiagen). The [γ−^32^P]ATP-labeled oligonucleotide was then annealed with a two fold excess of the unlabeled complementary oligonucleotide in annealing buffer (40 mM Tris-HCl, pH 8.0, 50mM NaCl) by heating at 95°C for 5 min and then cooling slowly to room temperature.

### Helicase assay

Equal amounts of SUMOylated BLM and unSUMOylated BLM, respectively, were added to 100 μl of nickel-NTA bead slurry (Qiagen) that had been blocked in 0.5% BSA for 2 h at room temperature and then washed two times in BLM binding buffer (60 mM Tris-HCl, pH 7.4, 20 mM KCl). BLM was bound to beads for 1 h at room temperature and washed two times in BLM binding buffer. Helicase reactions were performed with 20 ng BLM in 10 mM Tris-HCl (pH 7.5), 4 mM MgCl2, 1 mM dithiothreitol, 2 mM ATP, 0.1 mg/ml BSA, and 0.3 nM of labeled helicase substrate at 37°C for either 5, 10, 15, 20, 30, 40, or 60 min. Helicase reactions were stopped by adding gel loading dye (50 mM EDTA, 40% glycerol, 0.9% SDS, 0.05% bromophenol blue, and 0.05% xylene cyanol). Reaction products were analyzed by electrophoresis through pre-cast 10% non-denaturing polyacrylamide gels (Bio-Rad). The amount of substrate unwound was measured by autoradiography using a phoshorimager (Molecular Dynamics).

### *In vitro* binding assay

Two hundred nanograms of SUMOylated and unSUMOylated BLM, respectively, were added to 200 μl of Ni-NTA bead slurry (Qiagen) that had been previously incubated in Blocking Buffer (PBS, 0.5% Tween 20, 3% BSA) for 2 h at room temperature and subsequently washed three times with PBS. Increasing concentrations of purified RPA were then incubated with 20 ng of either SUMOylated BLM or unSUMOylated BLM bound to nickel-NTA beads, respectively, in binding Buffer (50 mM Tris-HCl, pH 7.4, 5 mM MgCl_2_, 5 mM ATP, 100 μg/ml BSA, and 50 mM NaCl) for 30 min at room temperature. Beads were then washed three times in binding buffer. The amount of RPA-70 protein bound to SUMOylated BLM or unSUMOylated BLM was analyzed by immunoblot with anti-RPA/p70 antibodies.

### Statistical analysis

Because observations within each clone may be correlated, we used mixed effects linear models to test the data for statistical significance. In the mixed effects models, each clone was treated as a random effect and the experimental variables were treated as fixed effects. For testing changes in the number of foci/cell, cell type (BLM or SM), treatment (with and without HU), and interaction terms for cell type by treatment were treated as fixed effects. Because the foci data were not normally distributed, we used a generalized estimating equation approach that can account for non-normal correlated data with non-homogeneous variances.

### Conflict of interest statement

The authors declare that the research was conducted in the absence of any commercial or financial relationships that could be construed as a potential conflict of interest.
