# Supplementary material for: BLM SUMOylation regulates ssDNA accumulation at stalled replication forks
Source: Front Genet. 2013 Sep 4;4:167. doi: 10.3389/fgene.2013.00167 (PMC3761158; doi:10.3389/fgene.2013.00167)
Supplement: Supplementary file 1 [file Presentation1.PPTX]

## Slide 1
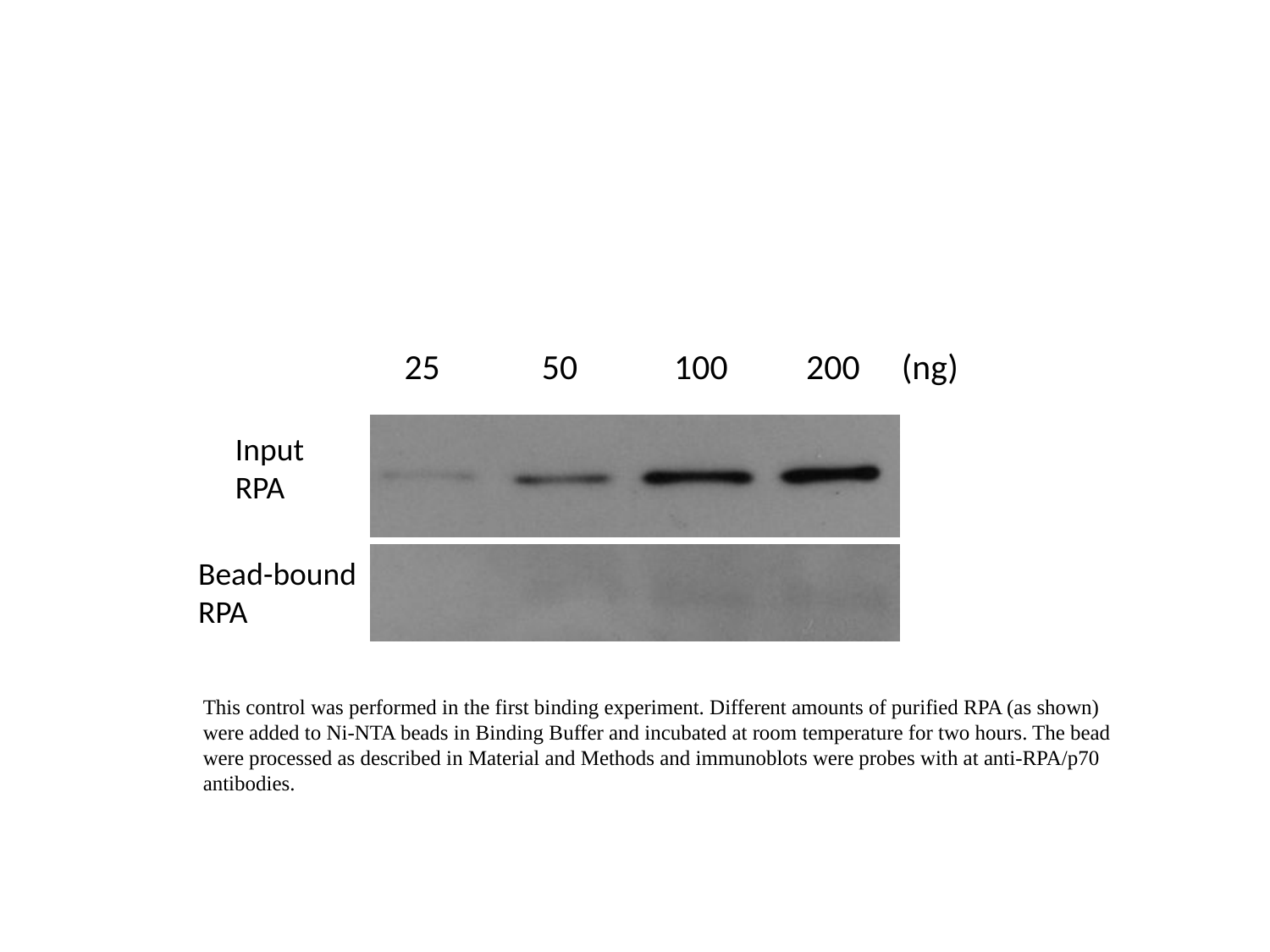

25
50
100
200
(ng)
Input
RPA
Bead-bound
RPA
This control was performed in the first binding experiment. Different amounts of purified RPA (as shown) were added to Ni-NTA beads in Binding Buffer and incubated at room temperature for two hours. The bead were processed as described in Material and Methods and immunoblots were probes with at anti-RPA/p70 antibodies.
